# Preparatory Medical School

**Published:** 1857-08

**Authors:** 


### PREPARATORY MEDICAL SCHOOL.

OUR attention has been directed to the Circular of Drs. Capers and Taliaferro of this city, who propose to establish a Preparatory Medical School in Atlanta.

As they remark, "Students generally enter College, unprepared to appreciate the opportunities offered for a complete medical education." This is indeed the crying evil connected with medical education. With proper preliminary education, before entering upon the study of the profession, and then a thorough training in a Preparatory Medical School, and a *faithful* adherence afterwards upon the part of the Colleges to the present three years study and two Courses of Lectures, we should have about all that is practicable in the present new and unsettled state of our country; this much, we think, can be accomplished, with earnest and *united* efforts for this *object;* but not even this will be attained, under the lead of visionaries and enthusiasts, or those who are keeping up an excitement upon this subject for selfish purposes.

Drs. Capers and Taliaferro propose giving instruction accompanied with daily recitations upon all the various branches usually taught in Medical Schools, for a term of five months, from 1st of November to 1st of April, including private instruction during the ensuing Session, in the Atlanta Medical College, making a term of nine months, for $50.

We can commend with the most entire sincerity and confidence, these gentlemen, as fully competent to the task which they propose, and earnestly desire to see their enterprise successful. With such a course of instruction as they propose, we are sure it would be worth more than thrice the time spent in the office of a private practitioner, in what is called "office study" which we all know, as a general rule, is a mere farce. As we learn from the Circular, the School has been provided with every facility for accomplishing the object of its establishment.

